# Upward Migration and Coiling of the Distal Catheter Toward the Valve Site

**DOI:** 10.7759/cureus.17993

**Published:** 2021-09-15

**Authors:** Khalid Alghamdi, Luma H Kutub, Ahmed G Qasem, Abdulrahman H Kaneetah, Sultan G Alzahrani, Hussam Y Kutub

**Affiliations:** 1 College of Medicine, King Saud bin Abdulaziz University for Health Sciences, King Abdullah International Medical Research Centre, King Abdulaziz Medical City, National Guard Health Affairs, Jeddah, SAU; 2 College of Medicine, Batargi Medical College, Jeddah, SAU; 3 Neurosurgery, King Fahad Hospital, Medinah, SAU; 4 College of Medicine, King Saud bin Abdulaziz University for Health Sciences, King Abdullah International Medical Research Centre, King Abdulaziz Medical City, National Guard Health Affairs, Jeddah , SAU; 5 College of medicine, King Saud bin Abdulaziz University for Health Sciences, King Abdullah International Medical Research Centre, King Abdulaziz Medical City, National Guard Health Affairs, Jeddah, SAU; 6 Neurosurgery, King Abdulaziz Medical City, Ministry of National Guards Health Affairs, Jeddah, SAU

**Keywords:** migration and coiling of ventriculoperitoneal shunt, ventriculoperitoneal shunt placement, pediatric hydrocephalus, upward migration of ventriculoperitoneal shunt, ventriculoperitoneal shunt complications

## Abstract

Hydrocephalus, which is caused by the accumulation of cerebrospinal fluid (CSF), is a common condition in children. It is known to be most likely treated by the insertion of a ventriculoperitoneal (VP) shunt. However, a VP shunt can lead to multiple complications. The upward migration of a VP shunt is considered rare.

A newborn male baby with a known case of Chiari malformation type 2 associated with myelomeningocele (MMC) and hydrocephalus had a VP shunt inserted for control of the hydrocephalus. He presented two months after the surgery with occipital swelling at the surgical site. Shunt series followed by Computerized tomography (CT) scan showed that the distal end of the catheter had migrated upward and coiled around the valve. Urgent revision of the VP shunt was performed.

Reabsorption of subgaleal fluid, increased abdominal pressure, repeated abdominal wall contraction, and repeated head motion of the child are the previously suggested theories of upward migration of distal catheter to the site of the valve. However, the combination of multiple theories can be the logical explanation, as they do not oppose each other.

## Introduction

Hydrocephalus is considered a common but serious condition in children caused by the accumulation of cerebrospinal fluid (CSF) [1]. The management of hydrocephalus is primarily surgical by insertion of a ventricular shunt [2]. The shunt diverts the accumulated CSF to another part of the body where it can be absorbed [2]. The most common type of shunt is a ventriculoperitoneal (VP) shunt [2]. However, several complications of the VP shunt have been reported, such as obstruction, over drainage of CSF, intracranial infection, seizure, abdominal injury, and visceral perforation [3]. The risk of complications from a VP shunt varies between 11% and 25% during the first year after surgery [4]. The subgaleal migration of VP shunt is considered a type of upward migration, and it is quite a rare complication [5]. We present a rare case of a patient with occipital swelling due to subgaleal upward migration of VP shunt, who was successfully treated. 

## Case presentation

**Figure 1:**
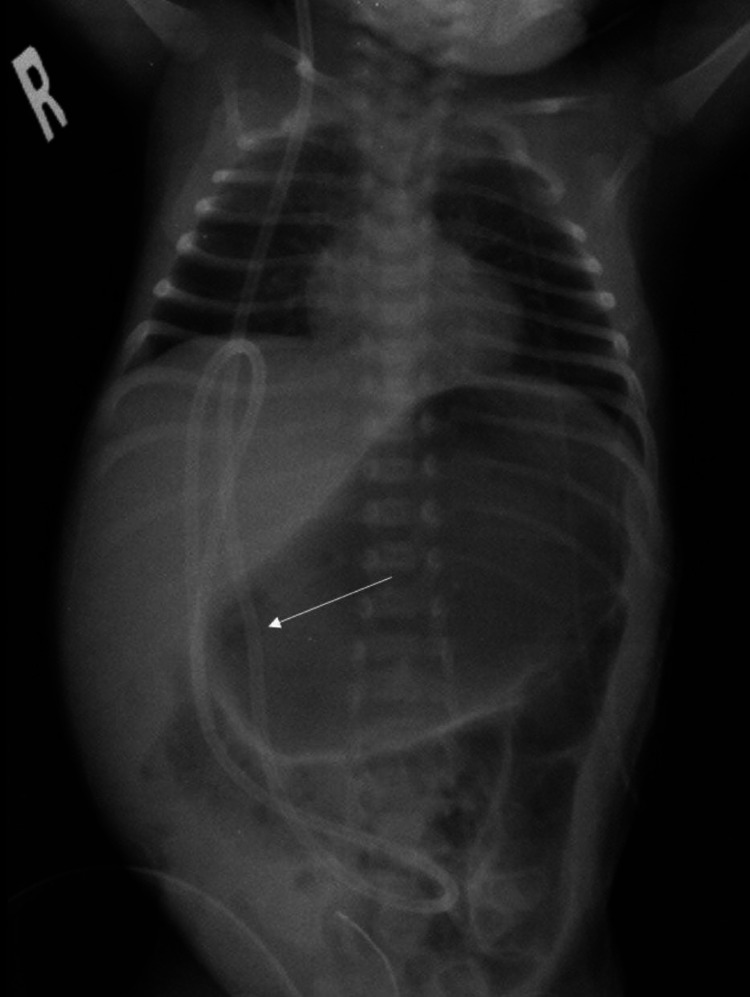
After the first operation, chest X-ray shows the extension of the catheter from the neck toward the peritoneal cavity.

A newborn male baby with a known case of Chiari malformation type 2 associated with myelomeningocele (MMC) had a repair of MMC after three days of delivery. The patient was then kept in a prone position. On the first day post MMC repair, his head circumference was 33.5 cm. Ten days post MMC repair, the patient was vitally stable and the fontanelle was soft and flat with head circumference increased by 2 cm (35.5), and computerized tomography (CT) scan of the brain showed interval increase in the size of the lateral ventricles. Hence, the patient underwent a VP shunt. During the surgery, a ventricular Bactiseal catheter was used and inserted to a depth of 4.5 cm, and CSF came in high pressure. The ventricular catheter was attached to a burr hole low-pressure valve. Adequate functioning of the shunt was confirmed, and there was good distal flow. The distal catheter was then inserted into the peritoneal region with sufficient length (20 cm). The patient was then transported back to the neonatal intensive care unit (NICU) in a stable condition (Figure 1).

**Figure 2:**
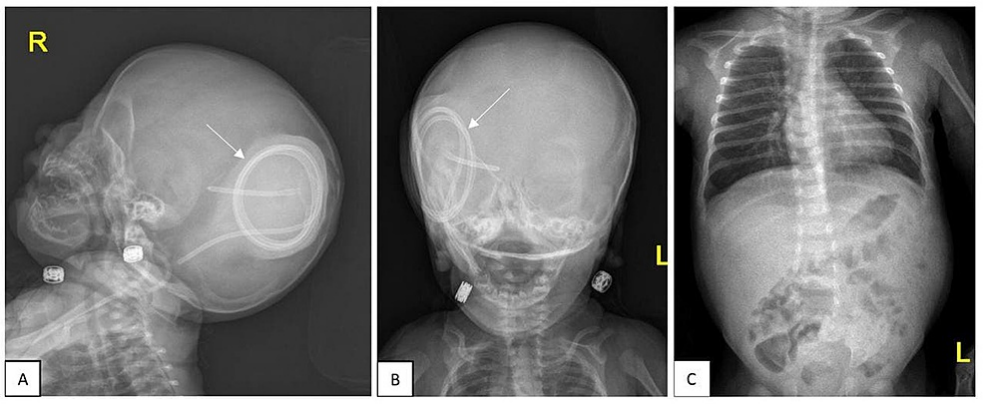
Before the second operation, VP shunt series, lateral (A) and anterior (B) skull view, show upward migration and coiling of the distal catheter. Chest X-ray (C) shows an absence of the distal catheter in the abdomen.

After one month, the patient presented to the neurosurgery clinic with swelling in the occipital region. Shunt series followed by CT brain showed extracranial CSF collection surrounding the valve of the right VP shunt with retraction and coiling of the distal catheter within the collection (Figure 2). Furthermore, an interval increase in the size of the lateral ventricles was noted, and the patient was booked for VP shunt revision the next day.

**Figure 3:**
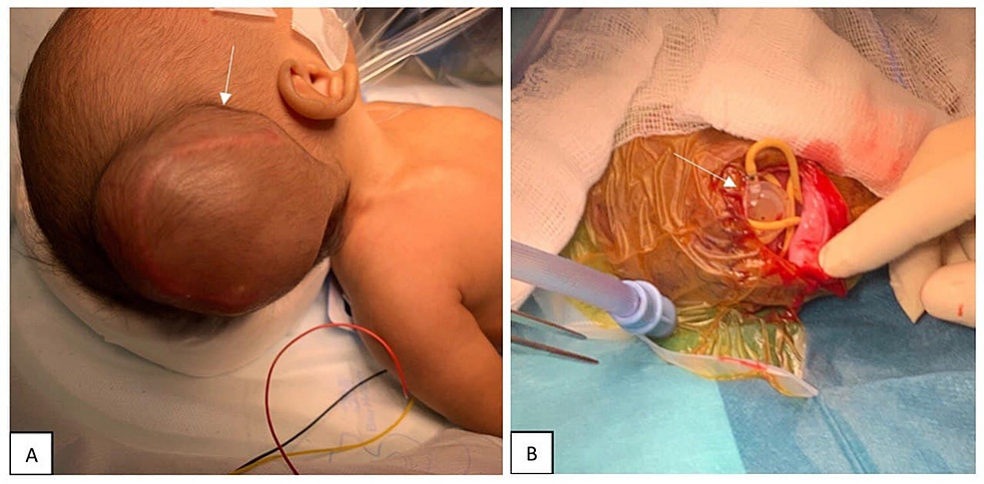
Images of the patient before incision (A) and after incision (B) show subgaleal swelling and coiling of the distal catheter at the valve site.

During shunt revision, the cranial incision was opened, and hypertensive flow was observed. The distal catheter was found coiled freely in the subgaleal space (Figure 3). Both the distal and proximal components of the ventriculoperitoneal shunt system were revised, and clear CSF was observed coming out from the distal catheter. The length of the distal catheter was increased and purse-string suture was used to prevent the recurrence of the same complication. Post VP shunt revision, the patient was vitally stable with a sunken anterior fontanelle.

## Discussion

The complications of shunt surgery can be divided into infection, functional failure, and mechanical failure which include disconnection, obstruction, and migration of any part of the shunt system [6]. Shunt migration seems to be more common in children than in adults because of the length of the distal catheter and repeated head motion of the child [7]. studies have shown migration of the distal catheter to multiple sites such as the heart, scrotum, chest, bladder, liver, colon, and anus. However, few studies reported cases of upward migration of the distal catheter of the VP shunt to the subgaleal space [6, 7, 8, 9, 10, 11]. The cases vary in their shunt system and whether they used the valve system or not, so the association between the device type and the development of upward migration was not found. Although subgaleal migration is a rare complication, many hypotheses have been reported, including reabsorption of subgaleal fluid, increased abdominal pressure, the repeated head motion of the child, and repeated abdominal wall contraction [8, 12, 13, 14]. In our case, the patient was kept in a prone position for 28 days after MMC repair, and that can lead to increase abdominal pressure. The increase in abdominal pressure has been explained by a study by Abou el Nasr, as it would lead to pushing force on the distal catheter [12]. Other hypothesis includes reabsorption of subgaleal fluid, which leads to the development of negative pressure that drags the distal catheter as explained by Pang et al. [13]. Scott et al. demonstrated " the windlass effect", which considers the repeated head motion of the child as the reason behind the upward migration of distal catheter [14]. The prevention of upward migration cannot be guaranteed, so securing a shunt device has been recommended [7]. In our case, a longer distal catheter (30 cm) was secured to the surrounding fascia to prevent the recurrent upward migration.

## Conclusions

The upward migration of the distal catheter to the valve site is considered rare. There are many speculated theories that can explain it. However, a combination of these theories may appear to be the logical explanation, as they do not oppose each other. Increased abdominal pressure and repeated head motion were considered to pull the distal catheter to the valve site at the subgaleal space. Further reporting of similar cases is required as it will increase the understanding of this complication.
